# Self-replication of circular DNA by a self-encoded DNA polymerase through rolling-circle replication and recombination

**DOI:** 10.1038/s41598-018-31585-1

**Published:** 2018-08-30

**Authors:** Yoshihiro Sakatani, Tetsuya Yomo, Norikazu Ichihashi

**Affiliations:** 1 Department of Bioinformatics Engineering, Graduate School of Information Science and Technology, Osaka University, 1-5 Yamadaoka, Suita, Osaka, 565-0871 Japan; 2 Institute of Biology and Information Science, East China Normal University, 3663 Zhongshan North Rd., Shanghai, 200062 P.R. China; 3 Graduate School of Frontier Biosciences, Osaka University, 1-5 Yamadaoka, Suita, Osaka, 565-0871 Japan

## Abstract

A major challenge in constructing artificial cells is the establishment of a recursive genome replication system coupled with gene expression from the genome itself. One of the simplest schemes of recursive DNA replication is the rolling-circle replication of a circular DNA coupled with recombination. In this study, we attempted to develop a replication system based on this scheme using self-encoded phi29 DNA polymerase and externally supplied Cre recombinase. We first identified that DNA polymerization is significantly inhibited by Cre recombinase. To overcome this problem, we performed *in vitro* evolution and obtained an evolved circular DNA that can replicate efficiently in the presence of the recombinase. We also showed evidence that during replication of the evolved DNA, the circular DNA was reproduced through recombination by Cre recombinase. These results demonstrate that the evolved circular DNA can reproduce itself through gene expression of a self-encoded polymerase. This study provides a step forward in developing a simple recursive DNA replication system for use in an artificial cell.

## Introduction

Recently, many researchers have proposed constructing artificial or minimal cell-like systems *in vitro* from biological molecules^1–5^. Reconstitution of cells and desired cellular functions from well-defined molecules enables us to understand the mechanisms or principles of target cellular functions^6–9^ and to develop new technologies^10–13^.

In all living organisms, genomes are replicated by DNA polymerase expressed from the genomes themselves via transcription and translation. Although expression of genome elements by cell-free systems has been examined^14–16^, *in vitro* reconstitution of a genomic DNA replication system is still an essential challenge for constructing artificial cells driven by a genome. To date, several *in vitro* DNA replication systems have been constituted, such as the genome replication systems of *Escherichia coli*^17,18^ and phi29 bacteriophage^19^, or artificial systems^20^; these replications were performed using externally supplied polymerase. Recently, Fujiwara *et al*. reported that 13 *E. coli* replication proteins are functionally expressed *in vitro*^14^. van Nies *et al*. reported linear DNA replication with four proteins from phi29 bacteriophage expressed in the same reaction mixture^21^. These reconstituted DNA replication systems were based on a natural genomic DNA replication schemes that requires 13 or four proteins. However, it is essential to design a simpler DNA replication system containing a smaller number of proteins. Constructing a simple DNA replication system is beneficial for creating an easily customizable artificial cell and can also provide insights into the possible origin of DNA replication on Earth.

A rolling-circle replication coupled with recombination was proposed as one of the simplest DNA replication systems^13^. As a first step to achieve this, we constructed a transcription and translation-coupled DNA replication (TTcDR) system containing a circular artificial genomic DNA encoding phi29 DNA polymerase^22^. In this TTcDR system, the entire sequence of the circular DNA was replicated in a rolling-circle manner by the phi29 DNA polymerase expressed from the DNA. However, this replication system was not recursive; the replication began with circular DNA but the product was linear DNA. To make this system recursive, circularization of the linear product DNA by Cre recombinase was proposed^13^. In our previous study, we attempted to introduce recombinase in the TTcDR system, but a circularized DNA did not form for unknown reasons^22^.

In this study, we found that one of the possible obstacles to circularization is that Cre recombinase inhibits rolling-circle replication. To overcome this problem, we used *in vitro* evolution to develop a circular DNA that can replicate efficiently in the presence of Cre recombinase. We found that one of the evolved DNAs reproduced circular DNA through rolling-circle replication and recombination.

## Results and Discussion

### Scheme of the TTcDR-recombination system

**Figure 1:**
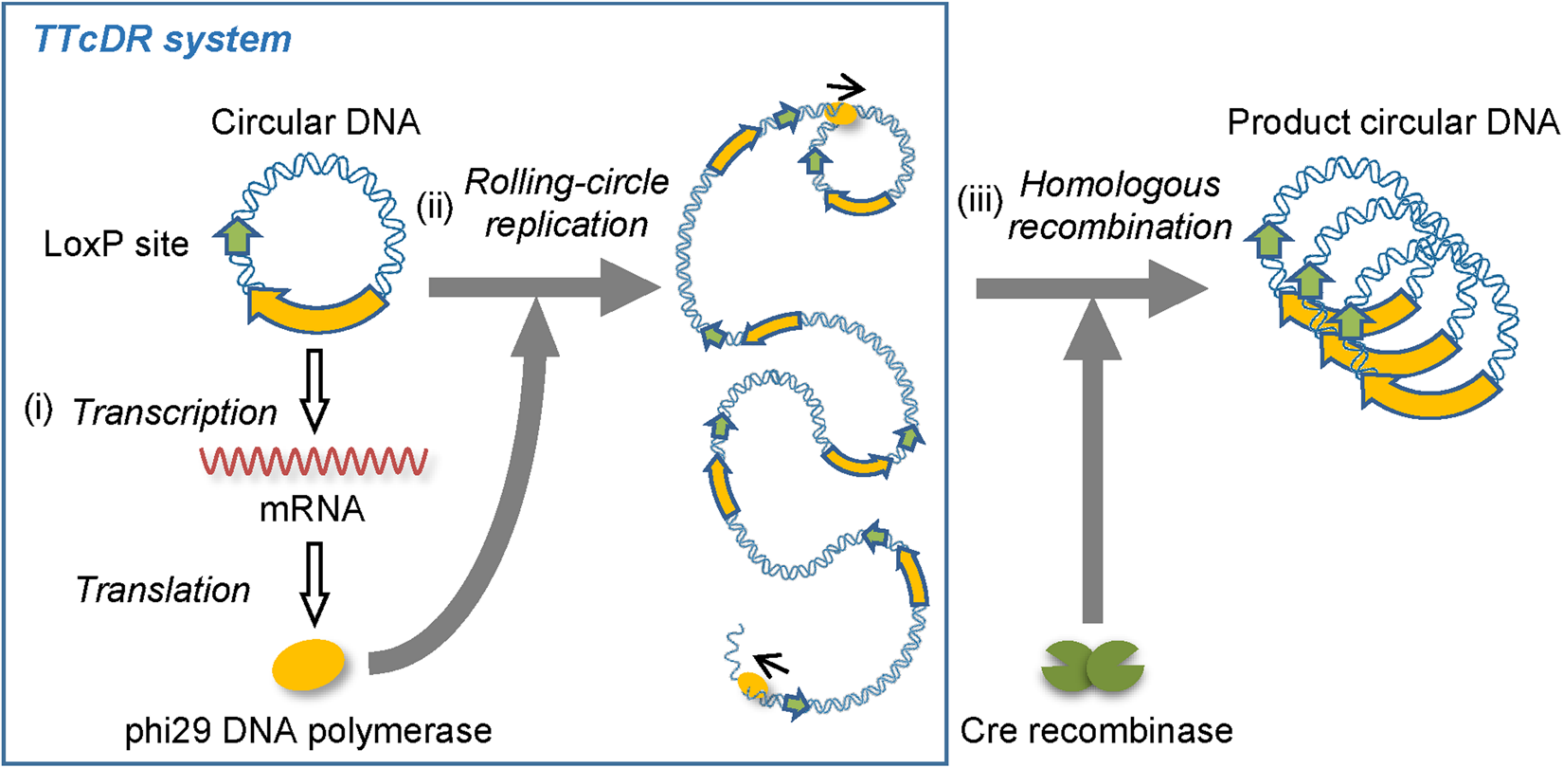
Scheme of the transcription and translation-coupled DNA replication (TTcDR)-recombination system to recursively replicate circular DNA. This system consists of a circular DNA containing the phi29 DNA polymerase gene and a loxP site and a reconstituted *Escherichia coli* translation system containing T7 RNA polymerase and Cre recombinase. (**i**) The DNA is transcribed to synthesize mRNA, which is then used to translate phi29 DNA polymerase. (**ii**) The translated polymerase initiates rolling-circle replication on the circular DNA to synthesize a long linear single-stranded DNA with tandem repeats of the original circular DNA sequence. The phi29 DNA polymerase also synthesizes the complementary strand of the single-stranded DNA to produce a linear double-stranded DNA. (**iii**) Cre recombinase recombines two loxP sites on the long DNA to produce a circular DNA product.

In this study, we attempted to establish a TTcDR-recombination system, the scheme of which is shown in Fig. 1. This system consists of a template circular DNA, a customized reconstituted translation system of *E. coli*^23^ including T7 RNA polymerase, and Cre recombinase. The template DNA encodes the phi29 DNA polymerase gene and the loxP site for recombination by Cre recombinase. In this system, (i) phi29 DNA polymerase mRNA is transcribed from the DNA by T7 RNA polymerase, and the phi29 DNA polymerase is translated from this mRNA. (ii) The translated polymerase initiates DNA polymerization using the circular DNA as a template to produce a long single-stranded DNA by rolling-circle replication because this polymerase has high processivity and strand-displacement activity. Phi29 DNA polymerase also synthesizes the complementary strand to produce a linear double-stranded DNA, a tandem repeat of the initial circular DNA sequence. These reactions are same as the TTcDR system reactions we reported previously^22^. (iii) Cre recombinase recognizes two loxP sites in the linear DNA and recombines these sites to reproduce a circular DNA. This is the new reaction introduced in the TTcDR system. This system does not contain any primers for DNA polymerization but DNA replication proceeds, probably because RNAs produced by T7 RNA polymerase function as primers^22^.

### Cre recombinase concentration required for efficient recombination

**Figure 2:**
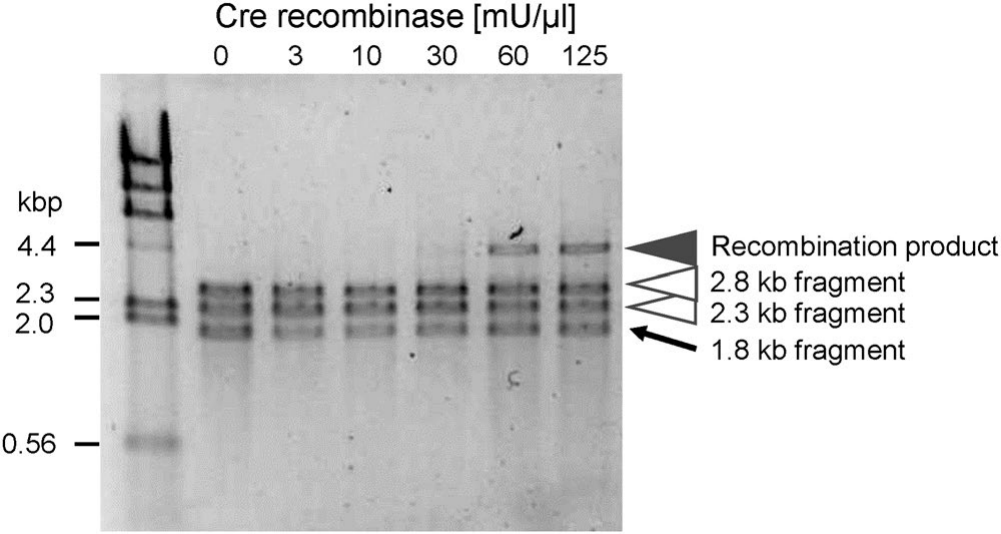
DNA recombination by Cre recombinase. Two DNA fragments (2.3 kb and 2.8 kb), each of which contains a loxP site, were incubated with the indicated concentrations of Cre recombinase, then subjected to 1% agarose gel electrophoresis and DNA staining with SYBR Green I. The black arrowhead indicates the recombination product. The bands around 1.8 kb (indicated with the arrow) represent a byproduct formed during DNA fragment preparation. The far left lane shows a size maker (λ-HindIII).

We first examined the Cre recombinase concentrations required for efficient recombination in the TTcDR reaction mixture. We prepared two linear DNA fragments (2.3 kb and 2.8 kb), each of which contained a single loxP site. A longer linear DNA fragment (4.7 kb) should appear after recombination between the loxP sites of these two fragments. We incubated the fragments at 37 °C for 4 h in a mixture identical to the TTcDR reaction mixture, omitting the nonessential components (i.e., tRNA, ribosome, and translation proteins). The longer DNA fragment was detected with more than 30 mU/μl Cre recombinase, and the amount reached a maximum with 60–125 mU/μl Cre recombinase (Fig. 2), indicating that 60 mU/μl of Cre recombinase is required to achieve maximum recombination efficiency in the TTcDR reaction mixture. The bands of around 1.8 kb (indicated with the arrow) represent a byproduct formed during the DNA fragment preparation.

### Identification of the TTcDR reaction step inhibited by Cre recombinase

**Figure 3:**
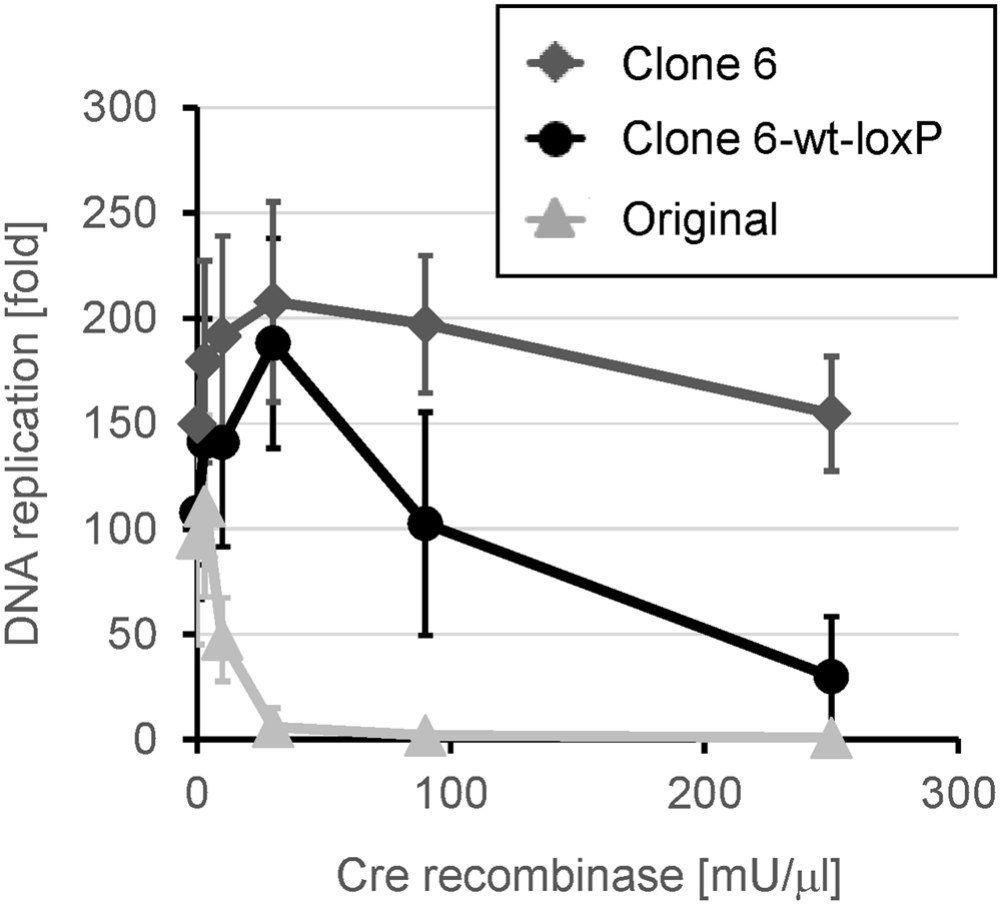
Effect of Cre recombinase on the transcription and translation-coupled DNA replication (TTcDR) reaction. The TTcDR reaction was performed with each circular DNA (0.40 nM) and the indicated concentration of Cre recombinase at 30 °C for 16 h. The amount of product DNA was measured by qPCR and the fold replication was calculated. The error bars indicate standard deviation (n = 3). The original DNA (Original) or the evolved DNAs (clone 6 and clone 6-wt-loxP) were used.

We next investigated the inhibitory effect of Cre recombinase on the TTcDR reaction using the original circular DNA. We performed the TTcDR reactions using various concentrations of Cre recombinase and measured the amount of product DNA. The DNA was amplified approximately 100-fold in the absence of recombinase, and replication decreased significantly in the presence of Cre recombinase at concentrations >10 mU/μl (Fig. 3, Original). This result indicated that Cre recombinase concentrations that allow the maximum recombination (i.e., more than 60 mU/μl, as shown in Fig. 2) significantly inhibit the TTcDR reaction.

**Figure 4:**
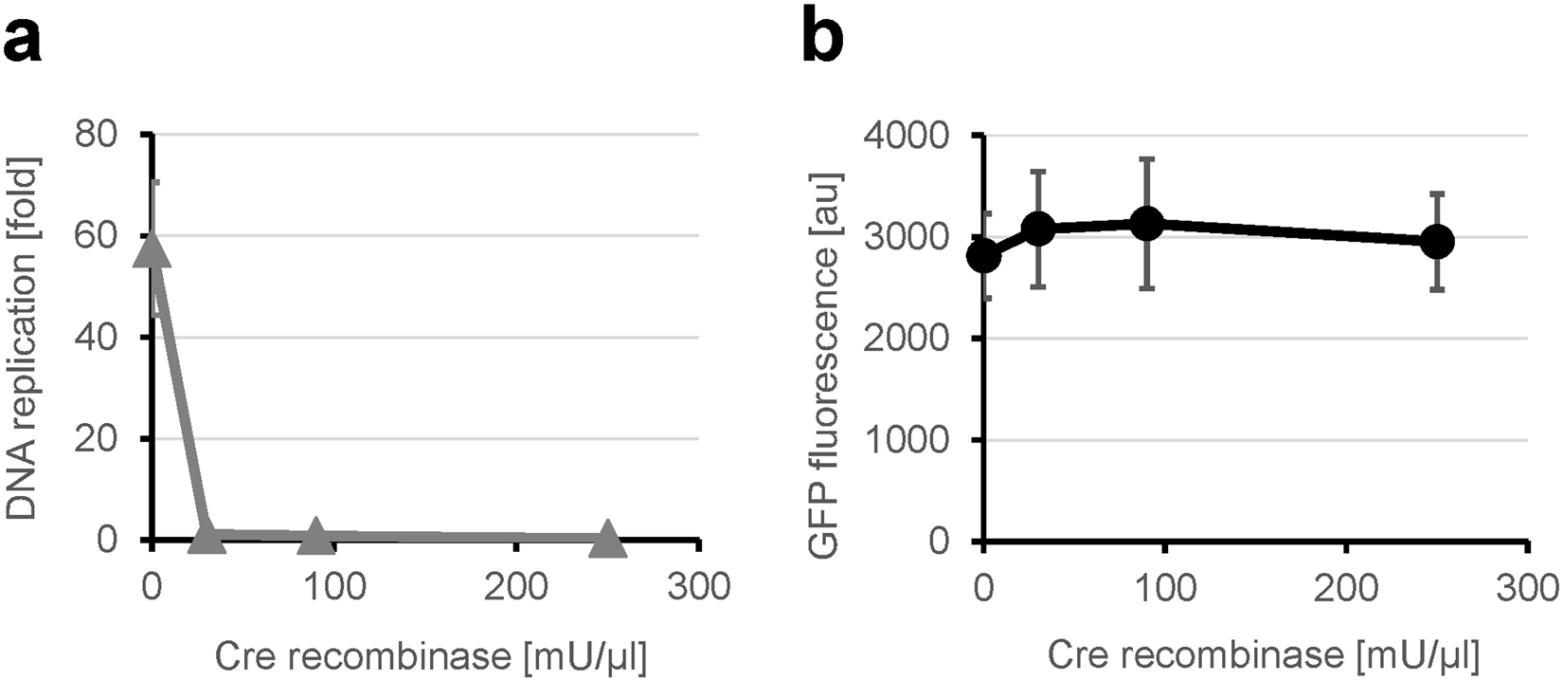
Effect of Cre recombinase on the polymerization by and the gene expression of phi29 DNA polymerase. (**a**) Effect of Cre recombinase on polymerization by phi29 DNA polymerase. DNA replication of the original circular DNA (0.71 nM) was performed using purified phi29 DNA polymerase (1 U/μl) and the indicated concentration of Cre recombinase in the standard buffer [0.3 mM each dNTP, 50 mM Tris-HCl (pH 7.8), 5 mM magnesium chloride, 7.5 mM potassium chloride, 0.1 mM dithiothreitol] at 30 °C for 12 h. (**b**) Effect of Cre recombinase on the gene expression of GFP. GFP was expressed using a DNA fragment encoding GFP under the T7 promoter (10 nM) and the indicated concentration of Cre recombinase in the TTcDR reaction mixture. Fluorescence intensity of GFP was measured after incubation at 37 °C for 2 h. The error bars indicate standard deviation (n = 3).

We further investigated the TTcDR reaction step inhibited by Cre recombinase using the original circular DNA. The TTcDR reaction can be divided into two steps: gene expression (i.e., transcription and translation) of the polymerase and the DNA replication by the polymerase. First, we studied the inhibitory effect of Cre recombinase on DNA replication by purified phi29 DNA polymerase with various concentrations of Cre recombinase. DNA replication proceeded up to 50-fold without Cre recombinase but was almost completely inhibited in the presence of more than 30 mU/μl Cre recombinase (Fig. 4a). Next, we studied the effect of Cre recombinase on gene expression by using the GFP reporter gene. The GFP fluorescence levels were similar for reaction mixtures with 0–250 mU/μl Cre recombinase. These results indicated that Cre recombinase inhibits the polymerization of the circular DNA catalyzed by phi29 DNA polymerase but does not inhibit gene expression.

### *In vitro* evolution to obtain Cre recombinase-resistant DNA

**Figure 5:**
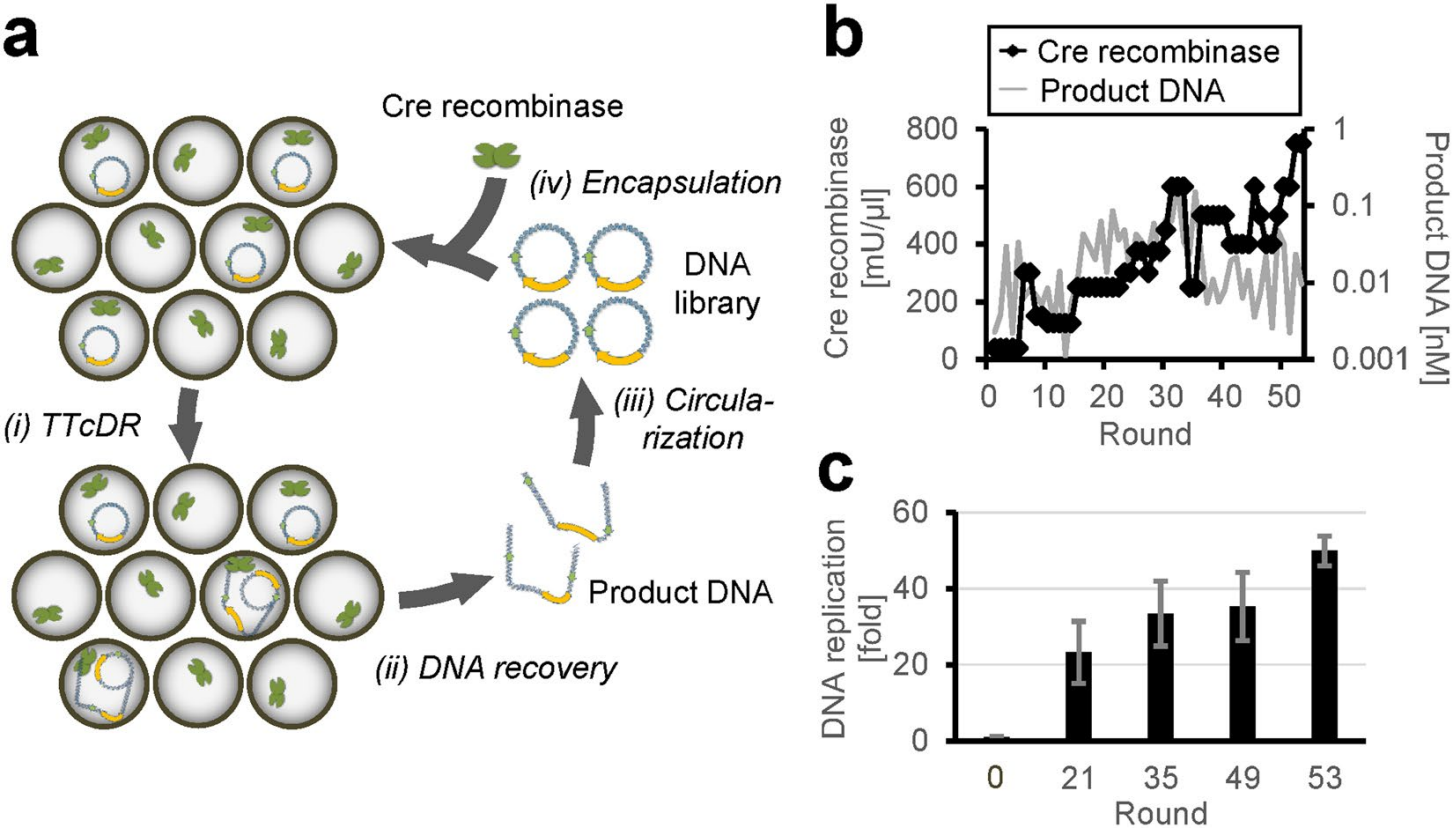
*In vitro* evolution of circular DNA that can replicate in the presence of Cre recombinase. (**a**) Experimental scheme. (i) The transcription and translation-coupled DNA replication (TTcDR) reaction of the circular DNA was performed in a water-in-oil droplet in the presence of Cre recombinase. The average number of circular DNA molecules per droplet was adjusted to be less than one. In the TTcDR reaction, phi29 DNA polymerase was expressed and it synthesized a linear DNA using the circular DNA as a template. (ii) The synthesized DNA was recovered from the droplets, and (iii) circularized with a ligase after PCR amplification, during which mutations were induced. (iv) The circularized DNAs were re-encapsulated into new water-in-oil droplets containing the Cre recombinase and the TTcDR mixture. The concentration of Cre recombinase was determined based on the concentration of the DNA product in the previous round; the recombinase was increased or decreased when the DNA product increased or decreased, respectively. (**b**) Trajectories of the Cre recombinase concentration and the average concentration of the product DNA. The product DNA concentration was measured by qPCR after step (i). (**c**) The average replication ability of the DNA populations at the indicated rounds in the presence of Cre recombinase. The TTcDR reaction was performed with the circular DNA population (0.40 nM) at each round in the presence of 250 mU/μl Cre recombinase at 30 °C for 16 h. The error bars indicate standard deviation (n = 3).

To overcome the inhibition of polymerization by Cre recombinase, we used an *in vitro* evolution experiment to develop a circular DNA that can replicate even in the presence of Cre recombinase. The evolutionary scheme is based on the “autogene” selection process^24^ and is schematically shown in Fig. 5a. We encapsulated the TTcDR mixture containing the Cre recombinase in water-in-oil droplets approximately 2 μm in diameter. The initial concentration of the circular DNA was 0.01 nM. (i) The TTcDR reaction was conducted by incubation at 30 °C for 16 h, and the product DNA was measured by quantitative PCR. (ii) The product DNA was recovered from the droplets. (iii) The product DNA was PCR-amplified followed by circularization through self-ligation. (iv) The circularized DNA was encapsulated again into droplets at the initial concentration for the next round of the TTcDR reaction. Mutations were introduced at approximately two mutations per round, mainly during PCR. If a mutant DNA that replicates more than other DNA molecules appears, the mutant DNA should dominate the population according to Darwinian principles.

The Cre recombinase concentration in the droplets was determined based on the average DNA replication rate of the previous round; the recombinase concentration increased or decreased when the average DNA replication rate was larger or smaller than that of the previous round, respectively. Therefore, if mutant DNAs that acquired resistance to Cre recombinase appear and dominate the population during the evolutionary cycle, the Cre recombinase concentration in the droplets should increase.

We repeated the evolutionary cycle for 53 rounds, and the amounts of the product DNA and the concentrations of Cre recombinase were plotted at each round (Fig. 5b). The Cre recombinase concentration at round 1 was 37.5 mU/μl, and it gradually increased as the rounds proceeded with a few fluctuations, and finally reached 750 mU/μl at round 53. To compare Cre recombinase resistance, we performed the TTcDR reaction of the circular DNA populations at rounds 0, 21, 35, 49, or 53 with 250 mU/μl Cre recombinase (Fig. 5c). The circular DNA at round 0 (original DNA) showed almost no replication, whereas the evolved DNA copy numbers increased as the rounds proceeded, and the final DNA population at round 53 was amplified more than 40-fold. This indicates that the circular DNAs acquired a certain level of resistance to Cre recombinase because of the *in vitro* evolution.

### Isolation of mutant DNAs at round 53

We isolated eight clones from the DNA population obtained after round 53 and compared their TTcDR reactions in the presence of 250 mU/μl Cre recombinase (Fig. S1). Six of the eight DNA clones showed a fold increase higher than that of the original DNA, and clone 6 replicated the most. These clones contained 17 mutations on average. Five mutations (A387G, C1640T, G1865A, G1903A, and G2100A) were common in more than 60% of clones, suggesting that these mutations contributed to the improved DNA replication. Three of them (A387G, C1640T, and G1865A) caused a change in the amino acid sequence of phi29 DNA polymerase, and the others (G1903A and G2100A) were present in the downstream region of the gene. In addition, three clones, including clone 6, showed a mutation C2022T in the loxP site. All the mutations of the isolated clones are listed in Table S1.

### Analysis of clone 6 and a derivative containing the wild-type loxP

In the remaining part of this study, we used clone 6 and its derivative (clone 6-wt-loxP). In clone 6-wt-loxP, the loxP site mutation (C2022T) was reverted from the clone 6 sequence because this mutation could hinder recombination by Cre recombinase.

We first compared the TTcDR reaction of these clones in the presence of various concentrations of Cre recombinase (Fig. 3). Clone 6 replication was slightly higher than that of the original DNA in the absence of Cre recombinase, and the replication did not decrease significantly even in the presence of 250 mU/μl Cre recombinase (Fig. 3, Clone 6). This result indicates that the clone 6 DNA acquired higher replication ability in the absence of Cre recombinase as well as a certain level of resistance to Cre recombinase. The replication of clone 6-wt-loxP was similar to that of clone 6 in the absence of Cre recombinase but was less than that of clone 6 in the presence of Cre recombinase, especially at high concentrations, indicating that the mutation in the loxP site (C2022T) partially contributes to Cre recombinase resistance.

The TTcDR activity of a circular DNA can be divided into two types: the ability to be polymerized as a template (template activity) and the ability to produce polymerase activity (polymerization activity). To investigate the template abilities of the evolved clones, we performed DNA replication using purified wild-type phi29 DNA polymerase in the absence and presence of Cre recombinase (30 mU/μl). The template activities of the evolved clones (clone 6 and clone 6-wt-loxP) decreased in the absence of Cre recombinase compared to that of the original clone, but were similar or higher in the presence of 30 mU/μl Cre recombinase (Fig. S2a). These results indicated that the change in the template activity partially contributed to the Cre resistance of clone 6.

We next assayed the polymerization activity of these clones by the following two-step reactions: i) the phi29 DNA polymerase was expressed from each clone in the absence of dNTPs to avoid DNA polymerization, and ii) replication of the circular DNA was performed in the absence or presence of 30 mU/μl Cre recombinase by the addition of dNTPs and a circular DNA. The DNA amplification amount was measured as an index of the polymerization activity. Both in the absence and presence of Cre recombinase, the polymerization activity was significantly increased in the evolved clones compared to that in the original DNA (Fig. S2b). This change in the polymerization activity also partially explains the improved Cre recombinase resistance of the evolved clones.

However, these template and polymerization activities cannot completely explain the difference in the TTcDR reaction between the original and the evolved clones shown in Fig. 3. For example, in the TTcDR reaction, the original DNA replication was significantly lesser than that of the evolved clones in the presence of 30 mU/μl Cre recombinase (Fig. 3), whereas the reduction levels in the template and the polymerization activities at 30 mU/μl Cre recombinase were not so low (Fig. S2). This suggests the involvement of another unknown mechanism for the Cre recombination resistance of the evolved clones.

### Examination of reproduction of circular DNA

**Figure 6:**
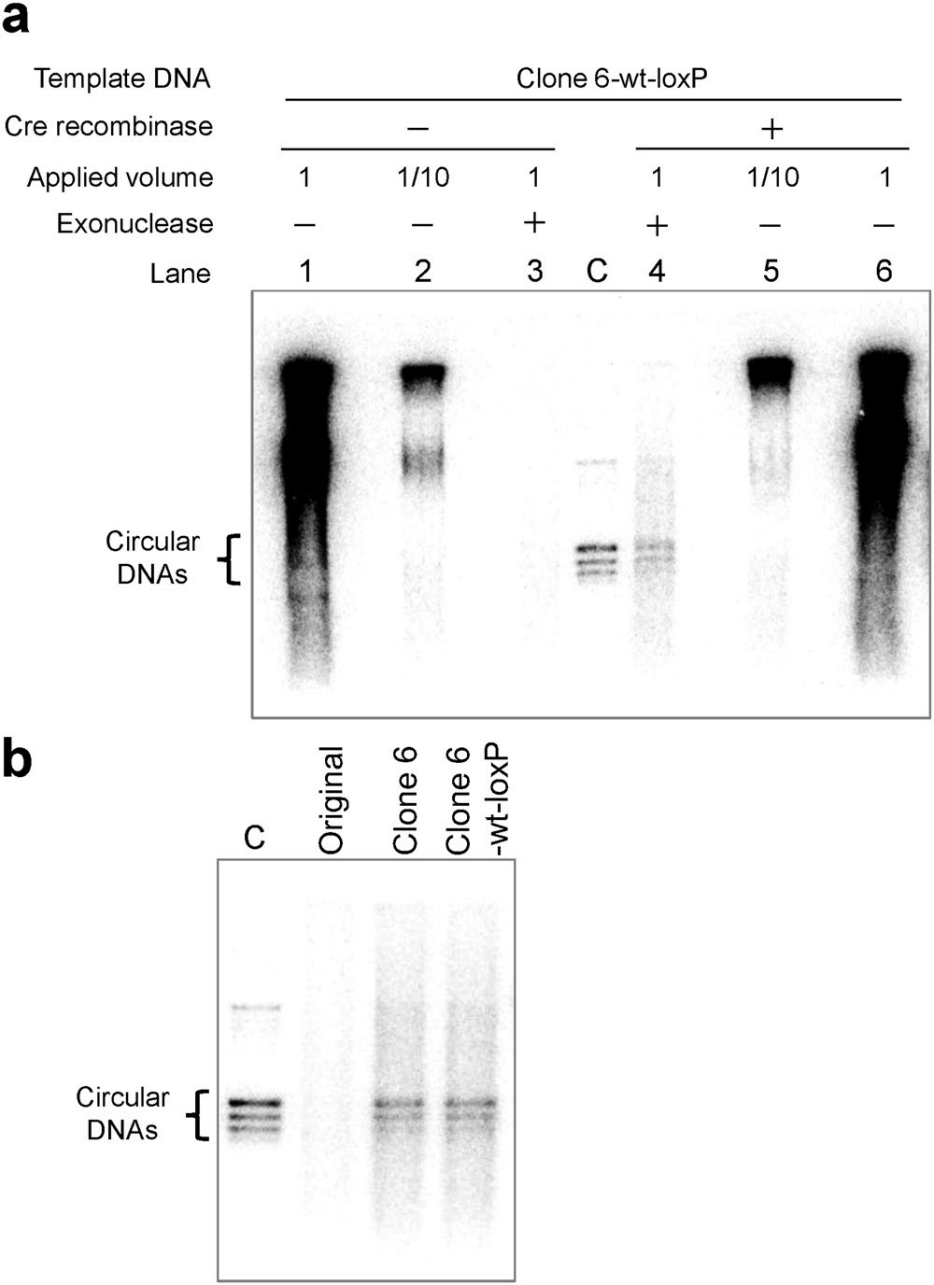
Electrophoresis of radiolabeled transcription and translation-coupled DNA replication (TTcDR) products. The TTcDR reaction was performed with [^32^P]-dCTP with or without 30 mU/μl Cre recombinase at 30 °C for 16 h. Before and after the degradation of linear DNAs with an exonuclease, the product DNA was subjected to 1% agarose gel electrophoresis, and [^32^P]-labeled DNA was detected by autoradiography. (**a**) Autoradiography of the TTcDR products of clone 6-wt-loxP (0.40 nM) with or without 30 mU/μl Cre recombinase before and after exonuclease treatment. Since the amount of DNA was too large before exonuclease treatment, we also applied 1/10 volume of the samples. The control circular DNA was applied in lane C. A lower contrast image is shown in Fig. S5. (**b**) Autoradiography of the TTcDR products of the original DNA, clone 6, and clone 6-wt-loxP. TTcDR reactions were performed in the presence of 30 mU/μl Cre recombinase and subjected to electrophoresis after exonuclease treatment.

To examine whether the circular DNA of clone 6-wt-loxP was reproduced during the TTcDR reaction coupled with recombination, we subjected the reaction mixture obtained after TTcDR reaction including radiolabeled dCTP with or without Cre recombinase (30 mU/μl) to agarose gel electrophoresis followed by autoradiography (Fig. 6a). Before treating the product with exonuclease, a large amount of DNA was detected in a broad range both in the absence and presence of Cre recombinase (lanes 1 and 6, respectively). One-tenth volume of samples was applied in lanes 2 and 5. After exonuclease treatment, which specifically degrades linear DNAs but not circular DNAs, no clear bands were detected in the absence of Cre recombinase (lane 3), whereas a few bands remained in the presence of Cre recombinase (lane 4) in the positions corresponding to the control circular DNA (lane C). These exonuclease-resistant, Cre recombinase-dependent bands were not detected when the TTcDR reaction was performed using the original circular DNA, but were detected when using evolved circular DNAs, clone 6 and clone 6-wt-loxP (Fig. 6b). These results suggest that circular DNAs were reproduced via the TTcDR-recombination reaction of the evolved clones. The amount of the circular DNAs was estimated approximately 3.6 nM based on the band intensities, suggesting that approximately 5% of the product DNA was circularized, corresponding to a 10-fold replication of the circular DNA.

In summary, these data support the interpretation that the bands (lane 4 of Fig. 6a, lands of clone 6 and clone 6-wt loxP of Fig. 6b) are formed by circular DNA. This interpretation is based on three facts: 1) the bands were exonuclease resistant, 2) the appearance of the bands depended on the presence of Cre recombinase, 3) the bands showed the same size and pattern as the control circular DNA.

The multiple bands of circular DNA can be attributed to variations in the topological winding number of a circular DNA because of three reasons. First, it is reported that circular DNA shows multiple bands caused by thermal fluctuations^25,26^. Second, the multiple bands were exonuclease resistant (Fig. S4). Third, digestion of the control circular DNA, with a single-cut restriction enzyme produced only a single band (Fig. S4).

To further confirm the reproduction of circular DNA, we transformed the product DNA into an *E. coli* strain. If the replicated DNA is circularized, it could replicate in the cell without being degraded. In this experiment, we used a plasmid containing each DNA sequence (original, clone 6, and wt-loxP derivative) and the ampicillin-resistant gene as a template circular DNA. We performed the TTcDR reaction with these plasmids in the absence or presence of Cre recombinase (30 mU/μl) for 0 h or 16 h. After degrading the initial methylated plasmid with the restriction enzyme DpnI, the replication products were introduced into an *E. coli* strain by chemical transformation. The *E. coli* strain was plated on agarose plates containing 50 μg/ml ampicillin and the number of colonies was counted after overnight incubation at 37 °C (Table 1).

**Table 1:** The number of colonies after transformation of the transcription and translation-coupled DNA replication (TTcDR) products.

Template DNA	Cre recombinase	Incubation time [h]*	The number of colonies (n = 3)**
Original	−	0	0 (0)
		16	3.3 (1.6)
	+	0	0 (0)
		16	9.3 (5.9)
Clone 6	−	0	0 (0)
		16	14 (6)
	+	0	0 (0)
		16	290 (110)
Clone 6-wt-loxP	−	0	0 (0)
		16	11 (10)
	+	0	0 (0)
		16	200 (74)

*The incubation time for the TTcDR reaction is indicated.

**Standard deviations are shown in the parenthesis.

For the samples not incubated in the TTcDR reaction (i.e., 0 h incubation time), no colonies were observed, whereas 3–290 colonies were observed after TTcDR reaction for 16 h, indicating that the colonies were products of the TTcDR reaction. More than one hundred colonies appeared for the evolved clones, clone 6 and clone 6-wt-loxP, in the presence of Cre recombinase, while the colony number was less than 15 when the TTcDR reaction was performed with original DNA or in the absence of Cre recombinase. These results suggest that a larger number of circular DNAs was produced in the TTcDR-recombination reaction for the evolved clones than for the original DNA, consistent with data shown in Fig. 6. Furthermore, these results indicate that the evolved clones, like vectors, can encode foreign genes (the ampicillin-resistance gene in this case) and replicate these genes together.

**Figure 7:**
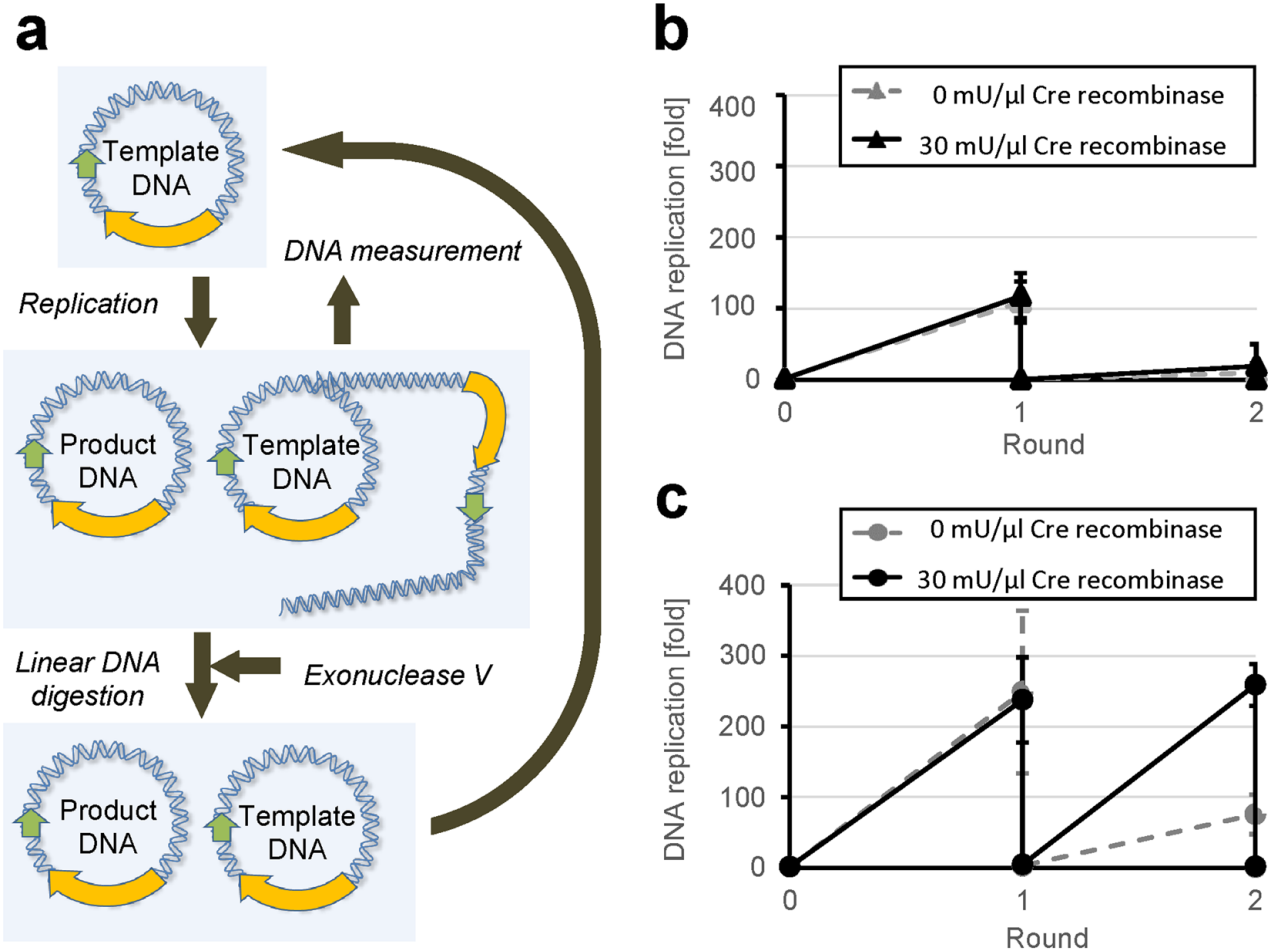
Replication of the reproduced circular DNA. (**a**) Experimental scheme. First, a circular DNA (0.40 nM) was replicated with or without 30 mU/μl Cre recombinase in the transcription and translation-coupled DNA replication (TTcDR) mixture at 30 °C for 16 h. Then, the linear DNA was digested with exonuclease V, and the residual circular DNA was used for the second round of the TTcDR reaction. qPCR was used to measure the DNA concentration after the TTcDR reaction before exonuclease treatment. (**b**) The DNA concentrations of the original DNA. The error bars represent standard deviation (n = 3). (**c**) The DNA concentrations of clone 6-wt-loxP. The error bars represent standard deviation (n = 3).

To further confirm that the circular DNA produced in the first round of replication can be used as a template for the next round of DNA replication, we purified the obtained DNA and performed the second round of replication with it. DNA. If a sufficient amount of circular DNA is reproduced, it will initiate the next round of the TTcDR reaction in the same way as the first round. After the first round of TTcDR reaction with 30 mU/μl Cre recombinase, we recovered the circular DNA products by degrading linear DNA with an exonuclease and used the remaining DNA to initiate the second round of the TTcDR reaction (Fig. 7a). In this experimental setup, at least 5-fold reproduction of the circular DNA was required in the first round to achieve the same level of replication in the second round.

For the original DNA, replication in the second round decreased significantly, compared to that in the first round, irrespective of the presence of Cre recombinase (Fig. 7b). In contrast, the replication of the evolved DNA (clone 6-wt-loxP) in the second round was the same as that in the first round only in the presence of Cre recombinase (Fig. 7c); this result is consistent with the detection of circular DNA in the presence of Cre recombinase shown in Fig. 6 and Table 1. This suggests that the circular DNA was reproduced during the first TTcDR reaction of the evolved clone. We also obtained a similar result with clone 6, although the amplification in the second round was slightly lesser than that in the first round (Fig. S3).

## Conclusion

In this study, we attempted to construct a simplified DNA replication system capable of reproducing a circular DNA by combining rolling-circle replication and recombination, according to the previous proposal^27^. By using the circular DNA clone obtained from *in vitro* evolution, we obtained evidence that the circular DNA replicated itself through a TTcDR-recombination reaction (Fig. 6 and Table 1) and that it can initiate the second round of a TTcDR reaction (Fig. 7c). These results indicate that the circular DNA developed in this study reproduced itself through rolling-circle replication and recombination. This system is one of the simplest isothermal DNA replication systems, requiring only two proteins, phi29 DNA polymerase and Cre recombinase, other than transcription and translation proteins. The results of this study provide an important step forward in synthesizing a simple artificial genomic DNA replication system.

The advantage of this DNA replication system is its simplicity; it requires only two proteins, polymerase and recombinase, for replication. Accordingly, it is easy to encode both proteins in the circular DNA. Presently, only the DNA polymerase was encoded and thus an important challenge to be addressed next is encoding of Cre recombinase on the circular DNA, which allows the development of a self-sustainable DNA replication system, a prerequisite for constructing an artificial cell.

## Materials and Methods

### DNA preparation

To prepare the original circular DNA, we first PCR-amplified DNA fragments encoding the phi29 DNA polymerase and loxP sequence using KOD FX (Toyobo, Japan), primer 1 (5′-CGGAGATCTCGTTGTAAAACGACGGCCAG-3′), primer 2 (5′-ACGAGATCTCCGGCTCGTATGTTGTGTGG-3′), and the template plasmid (pUC-phi29DNAP-loxP) constructed in a previous study^22^. The whole sequence of the original circular DNA is shown in Fig. S6. For clone 6 and clone 6-wt-loxP, corresponding plasmids, pUC-clone6 and pUC-clone 6-loxP-wt, respectively, were used. Preparation of these plasmids were described below. Then, we digested the DNA fragments with 0.5 U/μl BglII (TaKaRa, Japan) in the reaction mixture according to the manufacturer’s instruction for 1 h at 37 °C and self-ligated them using 1.75 U/μl T4 DNA ligase (TaKaRa) to produce the original circular DNA in the reaction mixture according to the manufacturer’s instruction for 1 h at 16 °C. For the circular DNA used for the *in vitro* evolution experiment, the initial PCR contained 0.12 mM alpha-S 2-deoxycytidine-5′-O-1-thiotriphosphate (dCTP; TriLink Biotechnologies). A DNA fragment encoding GFP under the control of the T7 promoter was prepared by amplifying the template plasmid pET-g5tag. We used PrimeSTAR HS (TaKaRa) and the primers 5′-GCGAAATTAATACGACTCACTATAGGG-3′ and 5′-GGTTATGCTAGTTATTGCTCAGCGG-3′ for amplification.

### Plasmids

The amplified DNAs, after 53 rounds of *in vitro* evolution experiments, were PCR amplified with primers, 5′-CGTTGTAAAACGACGGCCAG-3′ and 5′-CCGGCTCGTATGTTGTGTGG. They were then fused with a vector fragment and PCR amplified with primers, 5′-CGTCGTTTTACAACGTCGTGACTG-3′ and 5′-CAACATACGAGCCGGAAGCATAAAG-3′, using pUC19 as a template by using the In-Fusion HD Cloning Kit (Takara, Japan) according to the manufacturer’s instruction. These plasmids were named pUC-clone1 to pUC-clone6 according to the mutations shown in Table S1. The plasmid, pUC-clone6-loxP-wt, was prepared by reverting the mutation (C2022T) introduced in the loxP site of pUC-clone6 as follows: a DNA fragment was prepared by PCR amplification with primers, 5′-ATAGCATACATTATACGAAGTTATCCGCTGAGCAATAACTAGCG-3′ and 5′-TATAATGTATGCTATACGAAGTTATTGACAGCAGCCAACTCAGC-3′, using pUC-clone6 as a template and then fused by using the In-Fusion HD Cloning Kit.

### Enzymes

We followed the manufacturer’s instruction for defining the unit of each enzyme (RNA polymerase, RNase inhibiter, DNA polymerase, Cre recombinase, and so on).

### Recombination assay

We prepared DNA fragments, 2.3- and 2.3-kb or 2.8- and 1.8-kb fragments using PvuII- or EcoRI- (TaKaRa) mediated cleavage of the plasmid DNA (pUC-phi29DNAP-loxP), respectively. All of these fragments (5 nM) were incubated with various concentrations of Cre recombinase (New England Biolab) at 37 °C for 4 h in the TTcDR mixture lacking all proteins and translational RNAs. The reaction mixture was subjected to 1% agarose gel electrophoresis followed by staining with SYBR green I.

### DNA measurement

DNA concentration was measured by quantitative PCR (qPCR) using the original DNA as a standard. After each TTcDR reaction, reaction mixtures were diluted 100-fold with 1 mM EDTA (pH 8.0) and further diluted 5-fold with qPCR solution (SYBR Premix Ex Taq; TaKaRa) containing the primers 5′-AGGGTATGGGCGTATGGTTATATG-3′ and 5′-TGTCCCATGCGAGATATGATCG-3′. DNA fluorescence was monitored in real-time using Mx3005P (Agilent Technologies).

### TTcDR reaction system

The TTcDR reaction mixture contained 0.42 U/μl T7 RNA polymerase (TaKaRa), 0.1 U/μl RNase inhibitor (Promega), and the reconstituted translation system of *E. coli* as described previously^22^, except that the concentrations of dNTP and magnesium acetate were changed to 0.6 mM each and 10.5 mM, respectively. The protein components of this system were prepared by ourselves according to previously described methods^28^. The complete composition is shown in Table S2. In some experiments, the indicated concentrations of Cre recombinase were added in the mixture. In the experiment shown in Fig. 6, [^32^P]-dCTP (PerkinElmer) was included in the mixture. This system does not include any DNA primers because DNA replication began without DNA primers in this system, probably by using RNAs produced by T7 RNA polymerase as primers^22^.

### *In vitro* evolution experiment

The TTcDR mixture (10 μl) containing circular DNA (0.01 nM) labeled with α-S dCTP and Cre recombinase was mixed vigorously using a homogenizer in 1 ml buffer-saturated oil, as described previously^29^, to prepare a water-in-oil emulsion. The emulsion droplet sizes ranged from 1–4 μm diameter and the average was 2 μm^30^. After incubation at 30 °C for 16 h, the DNA concentration was measured by qPCR as described above. The emulsion was centrifuged at 20,000 × *g* for 5 min to collect the water droplets, and the droplets were washed with diethyl ether to remove the oil, as described previously^10^. The recovered water phase was mixed with 2 mM iodine solution and incubated at 37 °C for 5 min to degrade the initial DNA labeled with α-S dCTP. Then, 10 mM dithiothreitol was added, and the remaining DNA was PCR-amplified with KOD FX polymerase and primers 1 and 2. The PCR product was purified using a DNA column (PureLink PCR micro Kit; Life Technologies) and subjected to size selection using electrophoresis on a 1% agarose gel and the PureLink Quick Gel Extraction Kit (Life Technology). All DNA purification procedures in this study were performed using this column. The extracted DNA fragment was PCR-amplified again, as described above, except 0.12 mM α-S dCTP was added to label the DNA fragment. After purification, the DNA was digested with BglII and further purified. The purified DNA (0.71 nM) was self-ligated using T4 DNA ligase (1.75 U/μl) to produce a circular DNA, which was used for the next round of TTcDR reaction after purification.

### Template activity assay

Each circular DNA (0.56 ng/μl) was incubated at 30 °C for 16 h in the TTcDR mixture containing 1 U/μl of the purified phi29 polymerase, Cre recombinase (30 mU/μl), and streptomycin (30 ng/μl) to avoid polymerase expression in the reaction. The product DNA was measured by qPCR as an index of the template activity, as described above.

### Polymerization activity assay

First, linearized DNA molecules (0.56 ng/μl) of the original or evolved clones were incubated in the TTcDR mixture without dNTPs to avoid DNA polymerization at 30 °C for 4 h in the absence or presence of Cre recombinase (30 mU/μl). Second, a 1/6 (v/v) volume solution containing a dNTP mixture (0.6 mM each), the original circular DNA (0.56 ng/μl), and streptomycin (30 ng/μl) were added, and the mixture was incubated at 30 °C for 16 h to conduct the DNA polymerization by each polymerase expressed in the first reaction.

### Degradation of linear DNA by an exonuclease

Reaction mixtures were diluted 10-fold with deionized distilled water and mixed with 200 mU/μl exonuclease V (New England BioLabs), 1 mM ATP, and NEBuffer 4 (New England BioLabs, 50 mM potassium acetate, 20 mM Tris acetate, 10 mM magnesium acetate, 1 mM DTT, pH 7.9), and incubated at 37 °C for 4 h. After incubation, the mixtures were purified and concentrated 10-fold using a DNA column (PureLink PCR micro Kit).

### Electrophoresis followed by autoradiography

After the TTcDR reaction, the linear DNAs were degraded by the exonucleases as described above and subjected to 1% agarose gel electrophoresis with a control circular DNA. The gel was dried and subjected to autoradiography (FLA 7000, GE Healthcare). The control circular DNA was prepared using clone 6-wt loxP sequence as described above except that 3.3 µM [^32^P]-dCTP was included in the PCR step and degrading linear DNAs were incubated with exonuclease after self-ligation step. The results of agarose gel electrophoresis at each step are shown in Fig. S4.

### Transformation assay of the TTcDR products

The TTcDR reaction was performed using plasmids containing each DNA fragment (original, clone 6, clone 6-wt-loxP) and an ampicillin resistance gene in the presence or absence of 30 mU/μl Cre recombinase for 0 or 16 h at 30 °C. The reaction mixture was then diluted 20-fold and treated with 500 mU/μl DpnI (TaKaRa) at 37 °C for 2 h to degrade the initial plasmid DNA. The mixtures were purified and diluted 2.5-fold using a DNA column (PureLink PCR micro Kit). The purified DNA was transformed into an *E. coli* strain (DH5α) by a chemical method and spread onto a Luria-Bertani agar plate containing 50 μg/ml ampicillin. After 16 h of incubation at 37 °C, the number of colonies was counted.

## Electronic supplementary material

Supplemental figures

## Data Availability

All data generated or analysed during this study are included in this published article (and its Supplementary Information files).
