# Supplementary material for: Self-replication of circular DNA by a self-encoded DNA polymerase through rolling-circle replication and recombination
Source: Sci Rep. 2018 Aug 30;8:13089. doi: 10.1038/s41598-018-31585-1 (PMC6117322; doi:10.1038/s41598-018-31585-1)
Supplement: Supplementary file 1 — Supplemental figures [file 41598_2018_31585_MOESM1_ESM.pdf]

Supplementary information

Self-replication of circular DNA by a self-encoded DNA polymerase through rolling-circle replication and recombination

Yoshihiro Sakatani, Tetsuya Yomo, Norikazu Ichihashi

No.	Mutation	Change of protein	#1	#2	#3	#4	#5	#6	#7	#8
1	A51G						+			
2	A60G									+
3	G69C						+			
4	G98C						+			
5	G99A								+	
6	A100G		+	+	+			+		
7	A101G		+		+			+		
8	T102C					+				+
9	G104A						+			
10	A118T		+							
11	T119C		+							
12	T120C		+	+	+			+		
13	del T125			+	+					
14	T125C							+		
15	G308T	V51L				+				+
16	A354G	D66G							+	
17	A387G	N77S	+	+	+			+	+	
18	T398C	W81R				+				+
19	C646T		+							
20	A682G								+	
21	T685C						+			
22	G697A				+	+		+	+	
23	T700C				+	+		+	+	
24	G725A	A190T					+			
25	T748C		+	+						
26	G787A				+	+		+	+	
27	C795A	T213K	+	+						+
28	T814C						+			
29	G891A	G245D					+			
30	C898A		+	+						
31	T952C				+			+		
32	C1007T	H284Y					+			
33	G1015A		+	+						+
34	A1073G	R308G							+	
35	C1117T		+	+						
36	G1130A	D327N					+			+
37	C1177T							+		
38	A1285G					+				
39	G1409A	E418K				+				
40	A1454G	I433V	+							
41	A1552C						+			
42	A1558G	I467M					+			
43	C1640T		+	+	+	+		+	+	
44	C1654T		+							
45	C1666A	D503E							+	
46	T1767C	I537T					+			
47	G1771A									+
48	A1791G	N545S							+	
49	A1821C	K552T			+			+		
50	C1846T									+
51	G1865A	D567N	+	+	+	+		+	+	
52	G1903A		+	+	+	+		+		
53	A1940G						+			
54	del C1941-A1943			+	+			+		+
55	A1963G				+			+		
56	C1994T				+	+		+	+	
57	T1998C		+	+						+
58	T2020C								+	
59	C2022T				+	+		+		
60	T2027C								+	
61	G2033A			+						
62	C2070T								+	
63	G2100A		+	+	+	+	+	+	+	+
64	T2101C									+
65	A2111G				+			+		
66	del T2116								+	

Table S1. List of all the mutations in the eight clones at round 53.

The background color indicates the types of mutations: synonymous mutations (green), non-synonymous mutations (orange), and mutations in a loxP site (gray). Mutations written in red are common mutations that appeared in more than half of the eight clones. The whole sequence of the original DNA is shown in Figure S6.

Table S2. Composition of TTcDR system

Names of factors	Concentration	Names of factors	Concentration
Initiation factor 1	25 μM	valyl-tRNA synthetase	17 nM
Initiation factor 2	1 μM	methionyl-tRNA formyltransferase	590 nM
Initiation factor 3	4.9 μM	myokinase	1.4 μM
Elongation factor G	1.1 μM	creatine kinase	250 nM
Elongation factor Tu	80 μM	nucleoside diphosphate kinase	16 nM
Elongation factor Ts	3.3 μM	pyrophosphatase	41 nM
Release factor 1	49 nM	Trigger factor	1 μM
Release factor 2	48 nM	*E. coli* DEAH type RNA helicase A	100 nM
Release factor 3	170 nM	ribosomes	1 μM
ribosome recycling factor	3.9 μM		
alanyl-tRNA synthetase	730 nM	tyrosine	0.3 mM
arginyl-tRNA synthetase	31 nM	cysteine	0.3 mM
asparaginyl-tRNA synthetase	420 nM	18 other amino acids	0.36 mM
asparagyl-tRNA synthetase	120 nM	tRNA mix (Roche),	0.52 mg/ml
cysteinyl-tRNA synthetase	24 nM	ATP	0.375 mM
glutaminyl-tRNA synthetase	60 nM	GTP	0.25 mM
glutamyl-tRNA synthetase	230 nM	CTP	0.125 mM
glycyl-tRNA synthetase	86 nM	UTP	0.125 mM
histidyl-tRNA synthetase	85 nM	N-2-hydroxyethylpiperazine-N'-2-ethanesulfonic acid (pH 7.6)	100 mM
isoleucyl-tRNA synthetase	370 nM	glutamic acid potassium salt	70 mM
leucyl-tRNA synthetase	41 nM	spermidine	0.375 mM
lysyl-tRNA synthetase	120 nM	magnesium acetate	10.5 mM
methionyl-tRNA synthetase	110 nM	creatine phosphate	25 mM
phenylalanyl-tRNA synthetase	130 nM	dithiothreitol	6 mM
prolyl-tRNA synthetase	170 nM	5-formyl-5,6,7,8-tetrahydrofolic acid	10 μg/ml
seryl-tRNA synthetase	78 nM	each dNTP	0.6 mM
threonyl-tRNA synthetase	84 nM	yeast inorganic pyrophosphatase	0.2 mM
tryptophanyl-tRNA synthetase	28 nM	RNase inhibitor	0.1 U/μl
tyrosyl-tRNA synthetase	150 nM	T7 RNA plymerase	0.42 U/μl

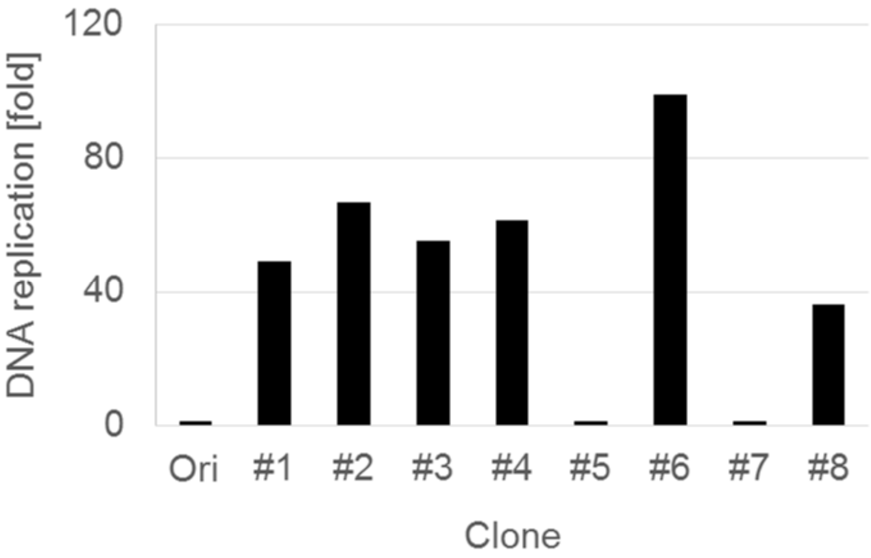

Figure S1. Evaluation of the eight clones at round 53.
The TTcDR reactions were performed with each DNA clone (1 ng/μl) at round 53 in the presence of 250 mU/μl Cre recombinase at 30 °C for 16 h, and the amount of product DNA was measured by qPCR. "Ori" indicates the original DNA before evolution.

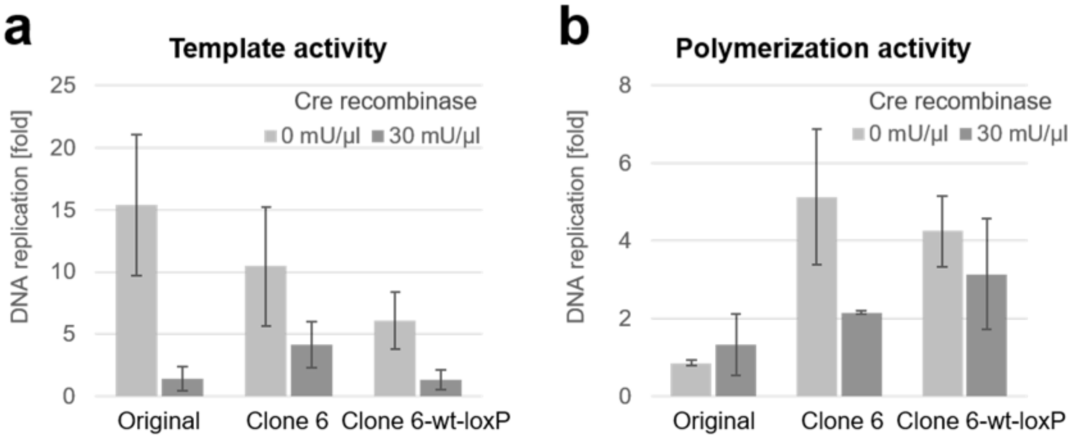

Figure S2. Template and polymerization activities of the evolved clones.
The template and polymerization activities of each clone with and without Cre recombinase were measured. The error bars represent standard deviations (n = 3).

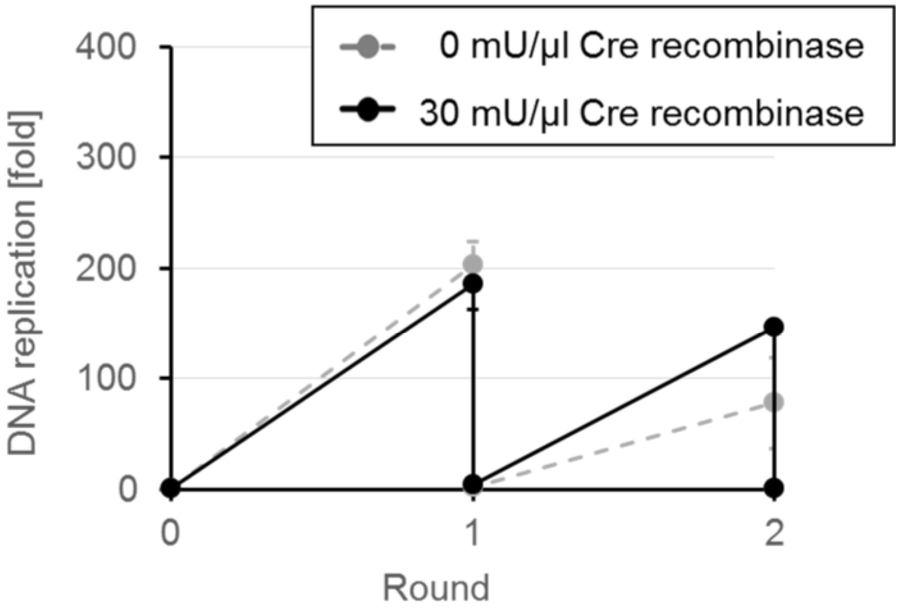

Figure S3. Replication of the reproduced circular DNA of clone 6.

The DNA concentrations in the first and second round of TTcDR reaction using clone 6 were measured as described in the legend of Fig. 7. The error bars represent standard deviations (n = 3).

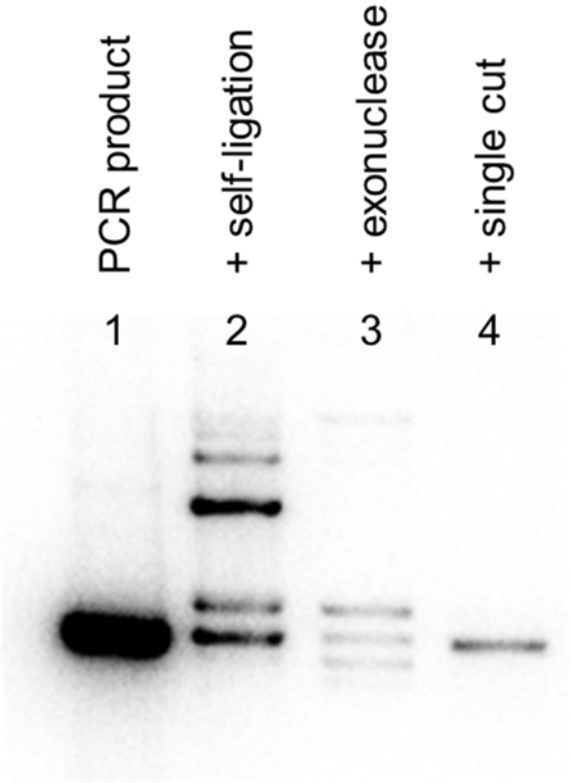

Figure S4. Examination of multiple bands of the control circular DNA

The control circular DNA used in Fig. 6 was prepared in three steps, 1) PCR amplification of clone 6-wt loxP sequence (lane 1, PCR product), 2) self-ligation of the PCR product (lane 2, + self-ligation), and exonuclease treatment to degrade linear DNAs (lane 3, + exonuclease). To confirm a single origin of the bands in lane 3, the DNA product after the exonuclease treatment was digested with a single cut restriction enzyme, BglII (lane 4, + single cut).

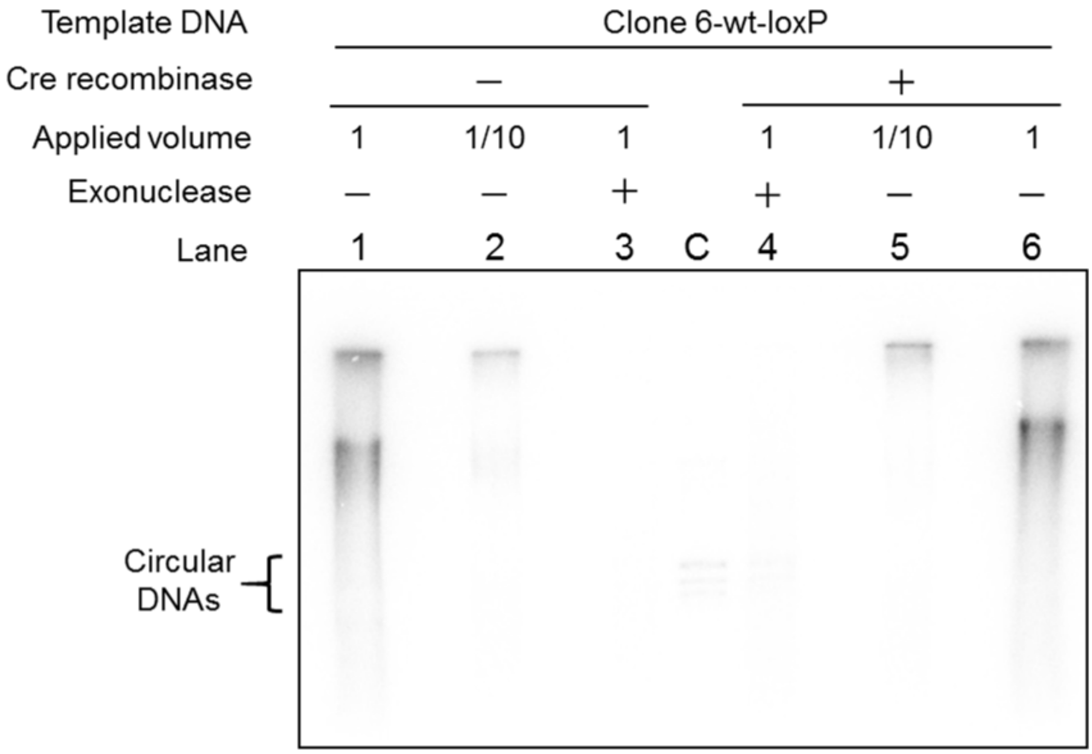

Figure S5. A lower contrast image of Figure 6a

agatctcgttgtaaaacgacggccagtgaattcgagctcggtacccggggatcctctagagtcgacctgcaggcatgctaatacgactcactata
ggggaattgtgagcggataacaattcccctctagaaataattttgtttaactttaagaaggagatatacatatgccgagaaagatgtatagttgtgact
ttgagacaactactaaagtggaagactgtagggtatgggcgtatggttatatgaatatagaagatcacagtgagtacaaaataggtaatagcctgg
atgagtttatggcgtgggtgttgaaggtacaagctgatctatatttccataacctcaaatttgacggagcttttatcattaactggttggaacgtaatggt
tttaagtggtcggctgacggattgccaaacacatataatacgatcatatctcgcatgggacaatggtacatgattgatatatgtttaggctacaaagg
gaaacgtaagatacatacagtgatatatgacagcttaaagaaactaccgtttcctgttaagaagatagctaaagactttaaactaactgttcttaaagg
tgatattgattaccacaaagaaagaccagtcggctataagataacacccgaagaatacgcctatattaaaaacgatattcagattattgcggaagct
ctgttaattcagtttaagcaaggtttagaccggatgacagcaggcagtgacagtctaaaaggtttcaaggatattataaccactaagaaattcaaaaa
ggtgtttcctacattgagtcttggactcgataaggaagtgagatacgcctatagaggtggttttacatggttaaatgataggttcaaagaaaaagaaa
tcggagaaggcatggtcttcgatgttaatagtctatatcctgcacagatgtatagccgtctccttccatatggtgaacctatagtattcgagggtaaat
acgtttgggacgaagattacccactacacatacagcatatcagatgtgagttcgaattgaaagagggctatatacccactatacagataaaaagaa
gtaggttttataaaggtaatgagtacctaaaaagtagcggcggggagatagccgacctctggttgtcaaatgtagacctagaattaatgaaagaac
actacgatttatataacgttgaatatatcagcggcttaaaatttaaagcaactacaggtttgtttaaagattttatagataaatggacgtacatcaagacg
acatcagaaggagcgatcaagcaactagcaaaactgatgttaaacagtctatacggtaaattcgctagtaaccctgatgttacagggaaagtccct
tatttaaaagagaatggggcgctaggtttcagacttggagaagaggaaacaaaagaccctgtttatacacctatgggcgttttcatcactgcatggg
ctagatacacgacaattacagcggcacaggcttgttatgatcggataatatactgtgatactgacagcatacatttaacgggtacagagatacctga
tgtaataaaagatatagttgaccctaagaaattgggatactgggcacatgaaagtacattcaaaagagctaaatatctgagacagaagacctatata
caagacatctatatgaaagaagtagatggtaagttagtagaaggtagtccagatgattacactgatataaaatttagtgttaaatgtgcgggaatgac
tgacaagattaagaaagaggttacgtttgagaatttcaaagtcggattcagtcggaaaatgaagcctaagcctgtgcaagtgccgggcggggtgg
ttctggttgatgacacattcacaatcaaataatgaattcgagctccgtcgacaagcttgcggccgcactcgagcaccaccaccaccaccactgaga
tccggctgctaacaaagcccgaaaggaagctgagttggctgctgccaataacttcgtatagcatacattatacgaagttatccgctgagcaataact
agcgcatgcaagcttggcgtaatcatggtcatagctgtttcctgtgtgaaattgttatccgctcacaattccacacaacatacgagccgg

Figure S6. Sequence of the original circular DNA

Phi29 DNA polymerase gene ranges from 167 to 1882. LoxP sequence ranges from 1996 to 2030.
